
JOURNAL INFORMATION
==============================
NLM Title Abbreviation: ScientificWorldJournal
Journal ID: ScientificWorldJournal
NLM Journal ID: 101131163
Journal ID: TSWJ

The Scientific World Journal

ISSN: 2356-6140
EISSN: 1537-744X
Publisher: Wiley

ARTICLE INFORMATION
==============================
PMCID: PMC6009490
PMID: 29973798
DOI: 10.1100/tsw.2002.24
Article ID: 619329
Article version: 1
Subjects: Short Report

Introducing New Amino Acids into Proteins

Schimmel Paul
Departments of Molecular Biology and Chemistry, Skaggs Institute for Chemical Biology, Scripps Research Institute, 10550 N. Torrey Pines Road, La Jolla, CA 92037, USA
Publication date: 2002
Electronic publication date: 2002 Mar 8
Volume: 2
First page: 47
Last page: 48
Copyright: Copyright © 2002 Paul Schimmel.
Copyright year: 2002
License: This is an open access article distributed under the Creative Commons Attribution License, which permits unrestricted use, distribution, and reproduction in any medium, provided the original work is properly cited.
License URL: https://creativecommons.org/licenses/by/3.0/

==============================
Advances in Gene Technology: The Genome and Beyond –
Structural Biology for Medicine (Proceedings of the 2002 Miami
*Nature Biotechnology* Winter Symposium)
*TheScientificWorld* 2002, 2(S2), 47–48
ISSN 1532-2246; DOI 10.1100/tsw.2002.24

### INTRODUCING NEW AMINO ACIDS INTO PROTEINS

Paul Schimmel
Departments of Molecular Biology and Chemistry, Skaggs Institute for Chemical Biology,
Scripps Research Institute, 10550 N. Torrey Pines Road, La Jolla, CA 92037

**INTRODUCTION**. The introduction of new amino acids into proteins presents a special challenge because, throughout the three kingdoms of life, the same 20 amino acids are used as the building blocks for all proteins. These building blocks have emerged from a long history of evolution when other amino acids were undoubtedly incorporated into early proteins. The use of the same amino acids in contemporary proteins is a direct result of the universal genetic code. The code, in turn, is established in the aminoacylation reactions catalyzed by aminoacyl transfer RNA (tRNA) synthetases. In these reactions, a specific amino acid is joined to the tRNA that bears the anticodon triplet corresponding to that amino acid. The tRNA synthetases themselves are ancient proteins whose evolution is thought to be closely tied to the development of the universal genetic code.

The introduction of new amino acids into proteins has at least two motivations. One is the practical value of producing proteins with novel activities or chemical properties that go beyond what is known for natural proteins. For example, proteins with novel catalytic groups, spectroscopic characteristics, or special surface properties could have a variety of applications. The other motivation is centered on the question of the origin of life and the transition from the putative RNA world to the theatre of proteins. This transition centers on the development of the genetic code and the appearance of proteins that may have contained amino acids other than or in addition to ones found in contemporary proteins. The early code probably was ambiguous, that is, a primitive codon could code for more than one amino acid. With these considerations in mind, efforts were directed at “invading” the genetic code so as to make possible the introduction of new amino acids into proteins. Because of their central role in establishing the code, a member of the aminoacyl tRNA synthetase set of enzymes was manipulated for these purposes.

**RESULTS**. Valyl-tRNA synthetase (ValRS) catalyzes the aminoacylation of $tRNA^{Val}$ in a two-step reaction. First, the enzyme condenses valine with ATP to yield a tightly bound valyl-adenylate. Next, the valyl adenylate is reacted with the 3’-end of $tRNA^{Val}$ to yield Val-$tRNA^{Val}$. In this way, valine is assigned to a specific nucleotide triplet (the tRNA anticodon) in the algorithm of the genetic code.

ValRS has two catalytic sites. One site is part of the catalytic center for the aminoacylation reaction. A pocket in the enzyme holds the isopropyl side chain of valine. However, the side chain of threonine, which replaces a single methyl group of valine with a hydroxyl group, can fit into the same pocket. As a consequence, ValRS will occasionally misactivate threonine and catalyze formation of Thr-$tRNA^{Val}$. Were it not for a second catalytic center, threonine would be inserted at valine codons. The second active site is a center for editing. As this active site misactivated amino acids are cleared, so that Thr-$tRNA^{Val}$ is broken down to release free threonine and $tRNA^{Val}$.

ValRS will activate noncanonical amino acids such as $\alpha$-amino butyrate (Abu), because its lacks one of the methyl side chains of valine and can fit into the binding pocket for valine. Here again, the editing center prevents stable attachment of Abu to $tRNA^{Val}$. However, the catalytic center for editing can be disrupted by mutation. As a consequence, mischarged $tRNA^{Val}$s can be generated. Using this approach, Abu was attached to $tRNA^{Val}$ and subsequently incorporated into cellular proteins *in vivo*. Through a single point mutation in the editing domain of ValRS, all cellular proteins were altered—up to 24% of the valines in *E. coli* proteins were replaced by Abu.

Thus, the genetic code can be “invaded” by disruption of the editing domain of a tRNA synthetase. As a consequence, a codon for a specific amino acid—valine—now is the signal for insertion of either valine or Abu. This circumstance, where a given codon will code for either of two sterically similar amino acids, may represent and early stage in the development of the genetic code. In this early stage, the code specified “rough” proteins, that is, where a specific protein was actually a heterogenous mixture of microspecies that had perhaps one of two or three possible amino acids at each position in the chain. Through the acquisition of the editing domain, the tRNA synthetases were able to perfect the aminoacylation reaction and give rise to the contemporary genetic code which lacks the sort of ambiguities that occurred when no such domain was present.

By disablement of the editing center, ambiguity was introduced into the genetic code so that Abu or Val were incorporated at valine codons. Further experiments can attempt to alter the catalytic center for aminoacylation, so that valine is now excluded and Abu is preferentially utilized. With the editing center simultaneously disrupted, Abu becomes the preferred amino acid to be inserted into proteins at codons for valine. Thus, these kind of approaches can be used to attempt to create homogenous proteins with one of the canonical amino acids replaced by a nonnatural amino acid.

**REFERENCES**

1. Lin, L., Hale, S.P., and Schimmel, P. (1996) Nature 384, 33–34.
2. Nomanbhoy, T., Hendrickson, T.L., and Schimmel, P. (1999) Mol. Cell 4, 519–528.
3. Döring, V., Mootz, H.D., Nangle, L.A., Hendrickson, T.L., de Crécy-Lagard, V., Schimmel, P., and Marlière, P. (2001) Science 292, 501–504.
